# Overexpression of soluble RAGE in mesenchymal stem cells enhances their immunoregulatory potential for cellular therapy in autoimmune arthritis

**DOI:** 10.1038/srep35933

**Published:** 2016-11-02

**Authors:** Min-Jung Park, Seung Hoon Lee, Su-Jin Moon, Jung-Ah Lee, Eun-Jung Lee, Eun-Kyung Kim, Jin-Sil Park, Jennifer Lee, Jun-Ki Min, Seok Jung Kim, Sung-Hwan Park, Mi-La Cho

**Affiliations:** 1 The Rheumatism Research Center, Catholic Research Institute of Medical Science, The Catholic University of Korea, Seoul 137-040, South Korea; 2 Divison of Rheumatology, Department of Internal Medicine, The Catholic University of Korea, Seoul 137-040, South Korea; 3 Department of Orthopedic Surgery, Uijeongbu St. Mary’s Hospital, College of Medicine, The Catholic University of Korea, Seoul, Korea

## Abstract

Mesenchymal stem cells (MSCs) are attractive agents for cellular therapy in rheumatoid arthritis (RA). The receptor for advanced glycation end products (RAGE) serves as a pattern recognition receptor for endogenous inflammatory ligands. Soluble RAGE (sRAGE) is a truncated form of RAGE that functions as a decoy and acts as an anti-inflammatory molecule. The aim of this study was to determine whether sRAGE has therapeutic effects and the mechanisms active in sRAGE-overexpressing MSCs (sRAGE-MSCs) in an experimental model of RA. sRAGE-MSCs were generated by DNA transfection of human adipose tissue-derived MSCs (Ad-hMSCs). MSCs showed increased expression of *VEGF*, *IL-1β*, *IL-6*, and *HMGB-1* under inflammatory conditions. However, sRAGE-MSCs showed significantly lower production of these proinflammatory molecules. Expression of immunomodulatory molecules such as IL-10, TGF-β, and indoleamine 2, 3-dioxygenase was higher in sRAGE-MSCs than in mock-MSCs. sRAGE-MSCs showed enhanced migration potential. Transplantation of sRAGE-MSCs into arthritic IL-1Ra-knockout mice markedly suppressed inflammatory arthritis, decreased Th17 cells, and reciprocally increased regulatory T cells. The differentiation of IFN-γ^+^CD4^+^ and IL-17^+^CD4^+^ cells was inhibited by incubation with sRAGE-MSCs compared with mock-MSCs. These findings suggest that sRAGE overexpression in Ad-hMSCs optimizes their immunoregulatory properties, which may be useful as a novel cellular therapy for RA.

Rheumatoid arthritis (RA) is a progressive autoimmune polyarthritis characterized by synovial hypertrophy and inflammatory cell infiltration into the affected joints because of an abnormal immune response that causes devastation of healthy joint tissues. The complex pathogenesis of RA involves many cell types, including CD4^+^ T cells, B cells, macrophages, and fibroblast-like synoviocytes in the inflamed hypertrophic synovium, or “pannus,” where these cells produce cytokines that perpetuate rheumatoid inflammation^1^. Accumulating evidence suggests that interleukin 17 (IL-17)- and IL-17-secreting CD4^+^ T (Th17) cells have pivotal roles in RA pathogenesis^2^. In contrast to Th17 cells, regulatory T (Treg) cells inhibit the activation and proliferation of immune effector cells by producing immunosuppressive cytokines such as IL-10 and transforming growth factor-β (TGF-β)^3^. Studies show that Treg cells isolated from RA patients have compromised immunosuppressive function compared with those from healthy people^4^,^5^, which suggests that reciprocal regulation of Th17 and Treg cells may be an ideal treatment strategy for human RA.

Currently, RA treatment remains a significant unmet medical need^6^ despite myriad therapeutic advances in biologics that have remarkable efficacy and acceptable safety profiles. Although biologics are more effective than synthetic disease-modifying antirheumatic drugs (DMARDs) in treating RA, a subset of patients achieves only partial remission^7^,^8^,^9^,^10^. Mesenchymal stem cells (MSCs) are cells of stromal origin that are present in various tissues including bone marrow, peripheral blood, umbilical cord blood, and adipose tissues. MSCs can exert profound immunoregulatory effects by modulating the proliferation and cytokine production of T and B cells, maturation of dendritic cells, and activity of NK cells^11^,^12^,^13^,^14^. Much recent research has focused on MSCs, and the results have encouraged the clinical application of MSCs in immunotherapy for autoimmune disorders including Crohn’s disease, type 1 diabetes, lupus erythematosus, and Sjögren syndrome^15^,^16^,^17^,^18^. MSCs can reduce the activity of Th17 cells and promote the differentiation of Treg cells^19^.

Although MSCs show beneficial effects in autoimmune disorders because of their anti-inflammatory activity, several preclinical studies have raised significant concerns about their therapeutic application in human RA. A recent study reported that intravenously infused MSCs induce inflammatory responses *in vivo*^20^. Moreover, MSCs fail to exhibit immunomodulatory properties when infused into the inflammatory micromilieu of autoimmune arthritis^21^. Therefore, the therapeutic potential of MSCs in experimental arthritis remains unclear^22^,^23^. Although MSC-based therapies have been effective to a certain degree, increasing evidence demonstrates that MSCs do not always achieve immunoregulatory function and that MSCs may promote immune responses only under certain conditions^24^. Therefore, optimization of MSC function may increase the opportunities for their clinical application in the treatment of autoimmune diseases including RA.

The receptor for advanced glycation end products (RAGE), a multiligand cell surface protein, is a member of the immunoglobulin (Ig) superfamily. RAGE participates in inflammatory and immune responses, inducing leukocyte recruitment. Soluble RAGE (sRAGE), a preventer of the RAGE signaling pathway, exerts anti-inflammatory activity by quenching RAGE ligands. sRAGE has been shown to reduce inflammation and to ameliorate several inflammatory diseases^25^,^26^. The blood levels of sRAGE are significantly lower in RA patients than in healthy controls, and synovial fluid from RA patients treated with DMARDs contained higher sRAGE concentrations compared with that from patients not treated with DMARDs, which shows compromised inflammatory control in active RA^27^.

Although autophagy induction in MSCs can optimize their clinical application by inhibiting the prevention of apoptosis^28^,^29^,^30^ and MSC treatment has potential therapeutic activity in RA^31^, MSCs fail to inhibit the immune modulation involved in the inflammatory condition of autoimmune arthritis^21^. MSCs in proinflammatory niches have immunoregulatory properties that can be induced by inflammatory cytokines such as tumor necrosis factor-α and establish an immunosuppressive microenvironment^32^. Thus, suppression of the MSC-induced inflammatory response is important for the use of MSCs in RA therapy because these cells are involved in the inflammatory response. It has been suggested that RAGE induces inflammatory responses by promoting HMGB-1^33^ and is expressed by MSCs^34^. Additionally, MSCs can be differentiated into cells that display a proinflammatory phenotype^35^. However, sRAGE can induce improvement of several inflammatory disorders^25^,^26^, and treatment with MSCs improves acute lung injury by decreasing the level of RAGE induced by lipopolysaccharide (LPS)^36^.

IL-1 receptor antagonist (IL-1Ra) prevents binding of IL-1, acts as an inhibitor of IL-1, and suppresses the progression of experimental arthritis^37^. The IL-1Ra-deficient strain derived from BALB/c mice spontaneously develops an excessive immune inflammatory response^38^ and increased frequency of Th17 cells^39^. IL-17 expression in T cells is increased by IL-1Ra deficiency^40^, and the unregulated IL-1 signaling mediated by IL-1Ra deficiency leads to the development of arthritis, excessive bone annihilation, and joint inflammation^41^.

To optimize the therapeutic potential of MSCs in RA, we hypothesized that sRAGE overexpression in adipose tissue-derived human MSCs (Ad-hMSCs) may augment their immunoregulatory properties in a murine model of RA. sRAGE was overexpressed by DNA transfection into Ad-hMSCs (sRAGE-MSCs). The anti-inflammatory, migratory, and differentiation potential of sRAGE-MSCswas quantified and compared with that of mock-MSCs. The *in vivo* immunoregulatory potential of sRAGE-MSCs was studied in IL-1Ra-knockout (IL-1Ra-KO) mice, an experimental model of RA. Finally, we evaluated the mechanisms underlying the augmented anti-arthritic effects of sRAGE-MSCs with respect to regulation of the Th17/Treg cell balance.

## Results

### Ad-hMSCs produce inflammatory mediators including high-mobility group box-1 (HMGB-1) when stimulated with LPS

**Figure 1:**
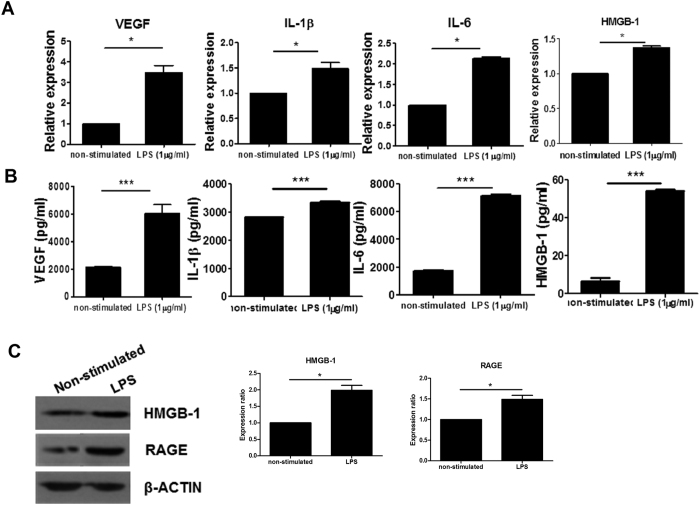
LPS-stimulated increase in the expression of proinflammatory factors in mesenchymal stem cells (MSCs). MSCs (2.5 × 10^5^) remained non-stimulated or were stimulated with lipopolysaccharide (LPS; 1 μg/mL) for 2 days. (**A**) mRNA expression of vascular endothelial growth factor (*VEGF*), interleukin-1β (*IL-1β*), *IL-6*, and high-mobility group box-1 (*HMGB-1*) was determined using real-time PCR. Data represent the mean ± s.d. (bars) values of three independent experiments. (**B**) VEGF, IL-1β, IL-6, and HMGB-1 concentrations in culture supernatants were measured using ELISA. (**C**) SDS-PAGE of protein lysates was followed by western blot analysis for HMGB-1, receptor for advanced glycation end products (RAGE), and β-actin. *P < 0.05 *vs*. non-stimulated MSCs.

Ad-hMSCs were first stimulated with LPS, and the mRNA expression of inflammatory mediators was measured and comparison with that of non-stimulated cells. The transcript levels of *VEGF*, *IL-1β*, *IL-6*, and *HMGB-1* in Ad-hMSCs increased significantly after LPS stimulation (Fig. 1A). Significantly higher concentrations of vascular endothelial growth factor (VEGF), IL-1β, IL-6, and HMGB-1 were found in the in culture supernatants from Ad-hMSCs treated with LPS compared with those from non-stimulated Ad-hMSCs (Fig. 1B). Western blot analysis showed that LPS stimulation increased the production of HMGB-1 and RAGE by Ad-hMSCs compared with the control unstimulated cells (Fig. 1C).

### Overexpression of sRAGE in Ad-hMSCs inhibits the expression of proinflammatory cytokines and increases the expression of immunomodulatory mediators

**Figure 2:**
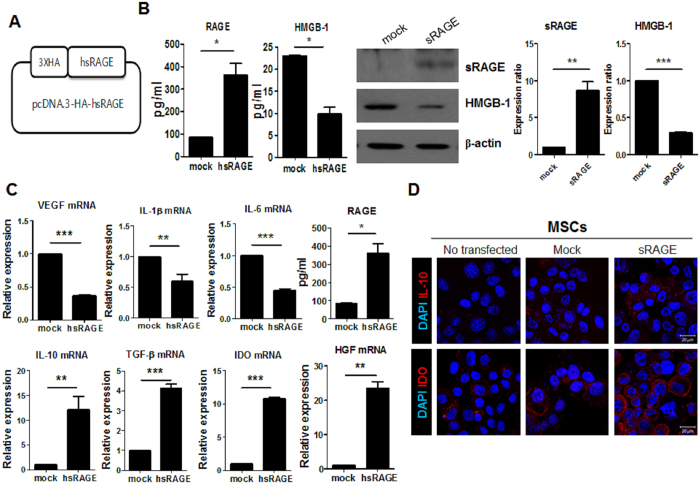
Reduced expression of proinflammatory factors in mesenchymal stem cells (MSCs) overexpressing the soluble receptor for advanced glycation end products(sRAGE). (**A**) Schematic representation of sRAGE DNA vector constructs. (**B**) MSCs were transfected with mock or sRAGE vector using the X-tremeGENE HP reagent for 3 days. RAGE and high-mobility group box-1 (HMGB-1) levels in MSCs and sRAGE-MSCs were measured by ELISA and western blotting. (**C**) Transcript levels of vascular endothelial growth factor (*VEGF*), interleukin-1β (*IL-1β*), *IL-6*, *HMGB-1*, *IL-10*, transforming growth factor-β (*TGF-β*), indoleamine 2,3-dioxygenase (*IDO*), and hepatocyte growth factor (*HGF*) were determined using real-time PCR. Data represent the mean ± s.d. (bar) values from three independent experiments. *P < 0.05. (**D**) Cultured MSCs and sRAGE-MSCswere stained for IL-10, IDO (red), and DAPI (FITC), and visualized by confocal microscopy.

We wanted to ascertain whether sRAGE overexpression could optimize the immunoregulatory properties of Ad-hMSCs. sRAGE was overexpressed in Ad-hMSCs by transfection of an sRAGE expression vector (Fig. 2A), which significantly decreased the expression and production of HMGB-1 (Fig. 2B). sRAGE overexpression also reduced the gene expression of proinflammatory cytokines such as *VEGF*, *IL-1β*, and *IL-6* in Ad-hMSCs (Fig. 2C, upper panel). The mRNA levels of immunomodulatory mediators including *IL-10*, *TGF-β*, *IDO*, and *HGF* were markedly higher in sRAGE-MSCs compared with mock-treated MSCs (Fig. 2C, lower panel). Confocal microscopy also confirmed the induction of IL-10 and indoleamine 2,3-dioxygenase (IDO) in sRAGE-MSCs (Fig. 2D).

### sRAGE overexpression in Ad-hMSCs enhanced their migratory capacity

**Figure 3:**
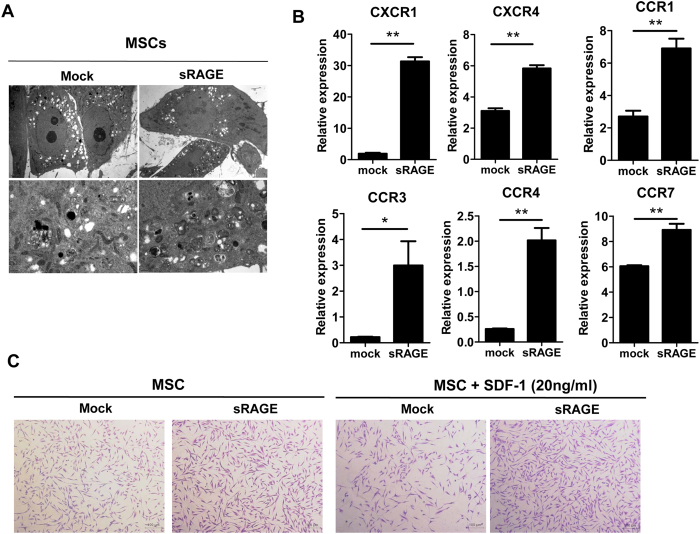
Cellular activity and migration of mesenchymal stem cells (MSCs) overexpressing soluble receptor for advanced glycation end products(sRAGE). (**A**) MSCs were transfected with the mock or sRAGE vector using the X-tremeGENE HP reagent for 3 days. Representative electron micrographs show the ultrastructure of mock- or sRAGE-transfected MSCs. (**B**) Transcript levels of chemokine (C–X–C motif) receptor 1 (*CXCR1*), *CXCR4*, chemokine (C–C motif) receptor 1 (*CCR1*), *CCR3*, *CCR4*, and *CCR7* were determined by real-time PCR. Data represent the mean ± s.d. (bars) values from three independent experiments. *P < 0.05. (**C**) Representative images of transmigrated MSCs (left) and sRAGE-MSCs (right) in response to stromal-derived factor-1 (SDF-1). MSCs were seeded in the upper chamber, and SDF-1 (30 ng/mL) was added to the bottom chamber of a 24-well Transwell unit (pore size, 8.0 μm).After 10 h, the migrated cells were stained with crystal violet. Scale bar = 100 μm.

Autophagy increases cell survival by inducing lysosomal degradation of damaged cytoplasmic organelles or cytosolic aggregates^42^. Several recent studies have revealed that autophagy induction in MSCs can optimize their clinical application by inhibiting the prevention of apoptosis^28^,^29^,^30^. Transmission electron microscopy revealed that sRAGE overexpression in Ad-hMSCs caused an increase in the appearance of double-membrane autophagic vacuoles (autophagosomes) in the cells compared with mock-MSCs (Fig. 3A). sRAGE-MSCs expressed significantly greater mRNA levels of chemokine (C–C motif) receptor 1 (*CCR1*), *CCR3*, *CCR4*, *CCR7*, chemokine (C–X–C motif) receptors(*CXCR1*), and *CXCR4*, which are involved in the migratory capacities of homing and tracking^43^ compared with the mock-treated Ad-hMSCs (Fig. 3B). The *in vitro* migratory capacity of sRAGE-MSCs toward the chemokine stromal-derived factor-1 (SDF-1) was also significantly higher than that of mock-MSCs (Fig. 3C).

### Overexpression of sRAGE does not affect the differentiation potential of Ad-hMSCs

**Figure 4:**
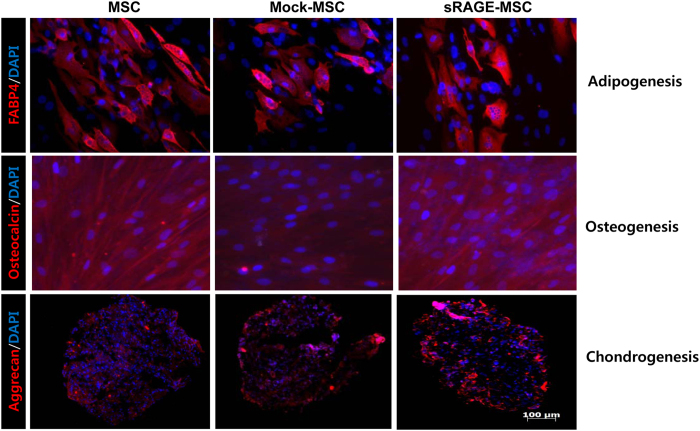
*In vitro* differentiation activity of mesenchymal stem cells (MSCs) overexpressing soluble receptor for advanced glycation end products(sRAGE). Mock- or sRAGE-transfected MSCs were cultured in each differentiation medium using a Human Mesenchymal Stem Cell Functional Identification Kit. After 2 weeks (adipogenic differentiation) or 3 weeks (osteogenic and chondrogenic differentiation), cells were fixed and stained with FABP-4 (top), osteocalcin (middle), or aggrecan (bottom), and visualized by fluorescence microscopy. Representative images of MSCs that differentiated into adipogenic, osteogenic, and chondrogenic lineages are shown.

To determine whether the overexpression of sRAGE in MSCs can affect their potential to differentiate into multiple cell lineages, MSCs were cultured in different conditions: adipogenic medium (Fig. 4, upper panel), osteogenic medium (Fig. 4, middle panel), or chondrogenic medium (Fig. 4, lower panel) for 2 or 3 weeks. MSCs, mock-MSCs, and sRAGE-MSCs showed similar patterns of differentiation into adipocytes, osteoblasts, and chondrocytes, which suggests that sRAGE-MSCs maintain their multipotency.

### sRAGE-MSCs exert greater therapeutic activity *in vivo*

**Figure 5:**
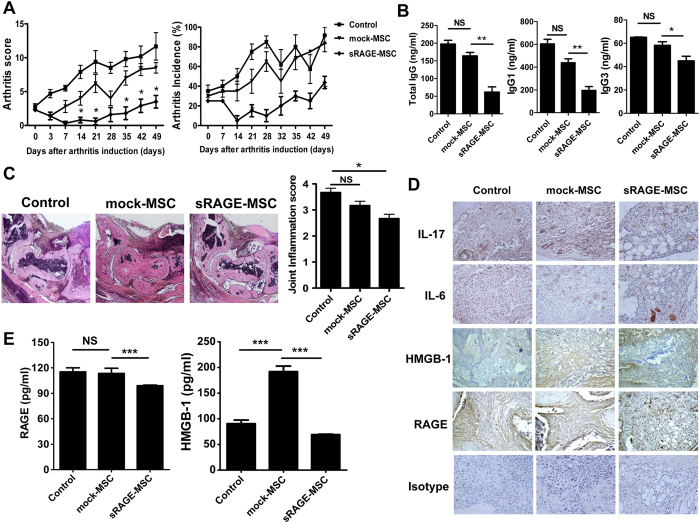
Attenuation of the severity of collagen-induced arthritis (CIA) by mesenchymal stem cells overexpressing soluble receptor for advanced glycation end products(sRAGE-MSCs). (**A**) CII-injected IL-1Ra KO mice were injected intravenously with 1 × 10^6^ MSCs (mock) or sRAGE-MSCsonce a week for 3 weeks after primary immunization. The clinical score (left) and incidence (right) of arthritis in treated mice are presented. Data are mean ± s.e.m. of arthritis scores of six mice per group. The incidence of arthritis is for animals with a score >4. (**B**) IgG, IgG1, and IgG3 antibodies in serum sample obtained 6 weeks after CII immunization (3 weeks after the injections with mock- or sRAGE-MSCs). Data represent mean ± s.d. (bars) of values derived from three independent experiments. *P < 0.05. (**C**) Hematoxylin and eosin (synovial inflammation, ×100)-stained representative joint sections from each group of mice, 6 weeks after primary immunization. Histological inflammation scores from each group are shown in (**B**). (**D**) Representative examples of immunohistochemical staining for interleukin-17 (IL-17), high-mobility group box-1 (HMGB-1), RAGE, and isotype controls in joint tissues from each mouse group. Positive immunoreactivity appears brown and is counterstained with blue (original magnification, ×400). (**E**) Three weeks after the injections, the levels of HMGB-1 and RAGE in serum were measured by ELISA. Data are presented as mean ± s.d. of three independent experiments. *P < 0.05.

We then investigated whether sRAGE-MSCs have increased immunomodulatory effects *in vivo*. The arthritis scores and arthritis incidence were compared between three groups: IL-1Ra-KO mice (control), mock-MSC-treated IL-1Ra-KO mice, and sRAGE-MSC-treated IL-1Ra-KO mice. sRAGE-MSCs exerted the greatest therapeutic effect *in vivo* compared with the control and mock-MSC-treated mice (Fig. 5A). Serum levels of total IgG, IgG1, and IgG3 were lower in mice treated with sRAGE-MSCs than in the other groups (Fig. 5B). Interestingly, mock-MSC-treated mice had serum autoantibody concentrations similar to those in the controls, which may explain the greater immunoregulatory potency of sRAGE-MSCs.

Histological examination showed that the paws and ankles from arthritis mice treated with sRAGE-MSCs exhibited the lowest degree of inflammation (Fig. 5C). Interestingly, compared with the controls, the expression of IL-17, IL-6, HMGB-1, and RAGE in inflamed joints was significantly higher in mock-MSC-treated IL-1Ra-KO mice and lower in joints of sRAGE-MSC-treated mice (Fig. 5D). Furthermore, the RAGE level in serum of controls was similar to that in mock-MSC-treated IL-1Ra-KO mice, whereas the concentrations of HMGB-1 in serum were significantly higher in mock-MSC-treated IL-1Ra-KO mice than in controls. (Fig. 5E). However, sRAGE-MSC treatment significantly reduced the expression of IL-17, IL-6, HMGB-1, and RAGE in inflamed joints and the concentrations of HMGB-1 and RAGE in serum.

### sRAGE overexpression in Ad-hMSCs reciprocally regulates the Th17 and Treg cell balance

**Figure 6:**
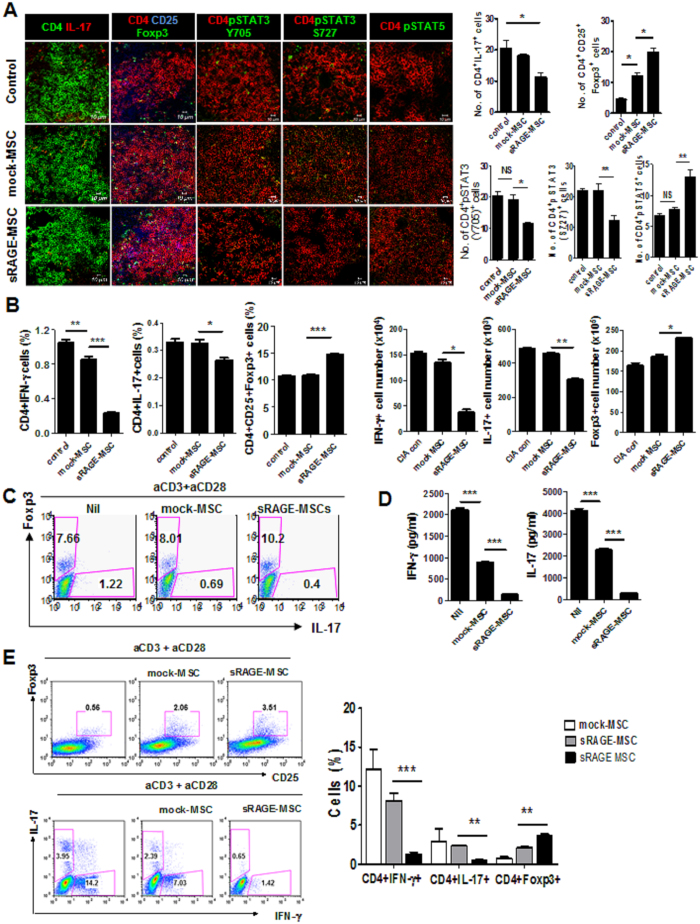
Regulation of CD4^+^ T helper cells by mesenchymal stem cells overexpressing soluble receptor for advanced glycation end products (sRAGE-MSC). (**A**) Type II collagen (CII)-injected IL-1Ra knockout (KO) mice were injected intravenously with 1 × 10^6^ MSCs (mock) or sRAGE-MSCsonce per week for 3 weeks after primary immunization. The expression of intracellular cytokine and transcription factors in splenic CD4^+^ T cells was determined by confocal microscopy at 6 weeks after primary immunization.Confocal microscopy images were obtained for each mouse (*n* = 6), and representative images are shown. (**B**) Splenocytes were isolated from each group and analyzed by flow cytometry for the expression of interleukin-17 (IL-17)- and interferon-γ (IFN-γ)-expressing CD4^+^ T and Foxp3^+^ Treg cells, respectively. (**C**) Activated mouse CD4^+^ T cells (anti-CD3 and anti-CD28 (0.5 μg/mL) for 3 days) were cocultured with MSCs or sRAGE-MSCs for 3 days. Cells were harvested and analyzed by flow cytometry for IL-17 and Foxp3 expression in CD4^+^ T cells. (**D**) IFN-γ and IL-17 concentrations in culture supernatants of mouse CD4^+^ T cells cocultured with mock MSCs or sRAGE-MSCs were measured by ELISA. (**E**) Activated human CD4^+^ T cells (anti-CD3 and anti-CD28 (0.5 μg/mL) for 3 days) were cocultured with MSCs or sRAGE-MSCs for 3 days. Cells were harvested and analyzed by flow cytometry for IL-17, IFN-γ, and Foxp3 expression in CD4^+^ T cells. (**F**) The concentrations of IFN-γ and IL-17 in the culture supernatant of human CD4+ T cells cocultured with mock MSCs or sRAGE-MSCs as determined by ELISA. Data are representative of at least three independent experiments. Bars represent mean ± s.d. of data from six mice per group. *P < 0.05.

T cells are implicated in the pathogenesis of RA, and IL-17 secreted by Th17 cells is a treatment target in RA. To identify whether Th17 and Treg cell populations were altered in IL-1Ra-KO mice treated with sRAGE-MSCs, IL-17-expressing (mainly Th17) and CD25^+^Foxp3^+^ (mainly Treg) cells among CD4^+^ T cells in spleens were analyzed by confocal microscopy at 7 weeks after CII immunization. Spleens from mice treated with sRAGE-MSCs had more Treg cells and reciprocally fewer Th17 cells compared with spleens from control mice and mock-MSC-treated mice (Fig. 6A). STAT3 and STAT5 are critical transcription factors for Th17 and Treg cell differentiation, respectively. As shown in Fig. 6A, spleens from sRAGE-MSC-treated mice has significantly fewer pSTAT3Y705 (phosphorylated at tyrosine 705)-expressing and pSTAT3S727 (phosphorylated at serine 727)-expressing cells compared with the other two groups. By contrast, spleens from sRAGE-MSC-treated mice had significantly more STAT5-expressing CD4^+^ T cells (Fig. 6A). Flow cytometry showed that spleens of sRAGE-MSC-treated mice had significantly fewer interferon-γ (IFN-γ)-expressing CD4^+^ T (Th1) cells and IL-17-expressing CD4^+^ T (Th17) cells but more CD4^+^CD25^+^Foxp3^+^ (Treg) cells (Fig. 6B) compared with the other groups.

To examine the immunoregulatory properties of sRAGE-MSCs related to the control of Th17/Treg cells *in vitro*, activated murine CD4^+^ cells were stimulated with anti-CD3 and anti-CD28 monoclonal antibodies (mAbs) (Th0 condition) and then cocultured with medium only (nil), mock-MSCs, or sRAGE-MSCs, and followed by flow cytometry to analyze the expression of Foxp3 and IL-17. sRAGE overexpression in Ad-hMSCs inhibited the Th17 population and reciprocally induced Foxp3+ Treg differentiation *in vitro* (Fig. 6C) compared with the nil condition and mock-MSCs. The concentrations of IFN-γ and IL-17 in the culture supernatants were lowest in murine CD4^+^ T cells cocultured with sRAGE-MSCs compared with the other two groups of T cells (nil and mock-MSCs) (Fig. 6D). Next, we confirmed the immunoregulatory effects of sRAGE-MSCs in human CD4^+^ T cells that had been stimulated under the Th0 condition (anti-CD3 and anti-CD28 mAbs). sRAGE-MSCs inhibited the differentiation of Th1 and Th17 cells from naïve T cells and reciprocally increased the Foxp3^+^ Treg cells (Fig. 6E).

## Discussion

Blockade of RAGE signaling can reduce inflammation, and sRAGE is a prominent target in the development of new treatments in inflammatory diseases. However, little is known about the biological roles of sRAGE in RA. Although MSCs are recognized as immunosuppressive agents in inflammatory disorders, they are also known to participate in the inflammatory process and in induction of immune responses^44^. Here, we investigated the therapeutic efficacy and underlying mechanisms of sRAGE-overexpressing MSCs in a murine model of inflammatory arthritis. The main observation of this study was that sRAGE-MSCs attenuated rheumatoid inflammation through reciprocal regulation of Th17 and Treg cells. To our knowledge, this is the first study to report data suggesting that sRAGE overexpression can optimize the immunotherapeutic potential of Ad-hMSCs; this may be a novel remedial strategy for the treatment of RA.

Although MSCs are considered to have potential for RA treatment by inhibiting the CII-reactive T cell responses^45^, naïve MSCs fail to elicit immunosuppressive and therapeutic functions *in vivo*^46^. It is well documented that the inflammatory micromilieu of RA can inhibit the therapeutic function of MSCs^21^. In our experiments, sRAGE-MSCs attenuated the clinical severity and histological changes of autoimmune arthritis, whereas naïve MSCs failed to show this effect. We also demonstrated that sRAGE overexpression in Ad-hMSCs maintained their migration and differentiation capacity. These data indicate that overexpression of sRAGE in Ad-hMSCs can refine their immunoregulatory potential while preserving their unique properties.

RAGE incites the inflammatory response by interacting with HMGB-1. Signal molecules whose release is initiated by RAGE bind to HMGB-1 and induce the expression of several proinflammatory cytokines^47^. HMGB-1 is a nonhistone nuclear protein that is ubiquitously present in eukaryotic cells^48^,^49^. It is released extracellularly by passive diffusion from necrotic cells and activates macrophages to trigger signals that drive the pathogenesis of autoimmune and inflammatory disorders by forming immunostimulatory complexes with cytokines and endogenous and exogenous factors^50^,^51^,^52^. The pathological role of HMGB-1 has been demonstrated convincingly. HMGB-1 levels in blood, synovium, and synovial fluids are increased in RA patients compared with OA patients and healthy people^53^,^54^,^55^. Animal studies have shown similar results^56^. HMGB-1 antagonists attenuate autoimmune arthritis in animal models, which supports the notion that HMGB-1 may be a treatment target in RA^57^. In our study, the serum and synovial concentrations of HMGB-1 were reduced in sRAGE-MSC-treated arthritic mice, whereas naïve MSC-treated mice showed significantly elevated HMGB-1 levels compared with vehicle-treated arthritic mice. These results may explain the lack of therapeutic efficacy of MSCs in RA.

Previous studies have shown that sRAGE inhibits the expression of inflammatory mediators *in vitro* and *in vivo*^58^,^59^. Moreover, administration of sRAGE exerts a therapeutic effect by downregulating chronic and severe inflammation in various animal models of inflammatory diseases such as atherosclerosis and inflammatory bowel disease^60^,^61^. A recent paper reported that sRAGE has therapeutic activity by attenuating the immune inflammatory response caused by HMGB-1^62^. The function of sRAGE is related to the inhibition of RAGE signaling in the inflammatory response^26^. In our investigation, sRAGE-overexpressing MSCs decreased the levels of inflammatory mediators and improved the symptoms of experimental arthritis. These results suggest that the therapeutic function of sRAGE-MSCs in arthritic mice can inhibit RAGE signaling in inflammatory conditions.

Several proinflammatory cytokines including IL-6, IL-1β, and VEGF are involved in RA pathogenesis and therapy^63^,^64^. However, IDO, IL-10, hepatocyte growth factor (HGF), and TGF-β exert immunosuppressive activity and cause an anti-inflammatory response^65^,^66^,^67^. Autophagy increases cell survival by inducing lysosomal degradation of damaged cytoplasmic organelles or cytosolic aggregates^42^. Several recent studies have reported that induction of autophagy in MSCs can optimize their clinical application by inhibiting the prevention of apoptosis^28^,^29^,^30^. In our study, overexpression of sRAGE in Ad-hMSCs decreased the expression of HMGB-1, IL-6, IL-1β, and VEGF, and increased the number of autophagosomes and IL-10, HGF, and TGF-β expression. These results suggest that sRAGE-MSCs can inhibit inflammation while promoting immunoregulatory responses. Thus, optimizing the immunoregulating capacity of sRAGE-MSCs may improve their therapeutic function in autoimmune arthritis.

Th17 and Treg cells are important T-cell subsets that can regulate RA pathogenesis. Th17 cells play a key role in inflammation of autoimmune arthritis^68^. The balance between Th17 and Treg cells is distorted in the peripheral blood of RA patients^69^, and Th17/Treg cell imbalance is a significant treatment target in RA. Several lines of evidence indicate that restoring Th17/Treg cell balance improves the severity of RA and reduces joint inflammation^70^,^71^,^72^. Here, we found that sRAGE-MSCs ameliorated the clinical severity of inflammatory arthritis in mice by regulating the Th17/Treg cell balance and the corresponding transcription factors. These results suggest that the therapeutic function of sRAGE-MSCs in arthritic mice may involve reciprocal regulation of Th17 and Treg cell differentiation.

Collectively, these results provide the first evidence of MSC optimization through sRAGE overexpression to suppress inflammatory disease. sRAGE-MSCs ameliorated the clinical severity of autoimmune arthritis by regulating the Th17/Treg cell balance and by inhibiting extracellular HMGB-1 activity compared with the activities of naïve MSCs. We conclude that this strategy to optimize the therapeutic potential of MSCs may help to control the inflammatory milieu of the rheumatoid synovium. The refined immunoregulatory function of sRAGE-MSCs identified in our research may have potential in the clinical applications of MSCs for cell therapy in RA.

## Materials and Methods

### Mice

IL-1Ra-KO mice with a BALB/c background were kindly provided by Prof. Yoichiro Iwakura (University of Tokyo, Japan) and were maintained under specific pathogen-free conditions at the Institute of Medical Science, Catholic University of Korea. Standard mouse chow (Ralston Purina, St Louis, MO, USA) and water were provided ad libitum. All experimental procedures were examined and approved by the Animal Research Ethics Committee of The Catholic University of Korea (2014-0078-02), in conformity with the National Institutes of Health guidelines.

### Induction of arthritis and treatment with sRAGE-MSCs

To augment the arthritis severity, 0.1 mL of an emulsion containing 100 μg of bovine CII and complete Freund’s adjuvant (Arthrogen-CIA; Chondrex, Seattle, WA, USA) was injected intradermally into the base of the tail. After CII immunization, mice were injected intravenously with 1 × 10^6^ Ad-hMSCs or sRAGE-MSCs once per week for 3 weeks. Clinical signs of arthritis in IL-1Ra-KO mice were monitored visually and scored twice per week as reported previously^73^. The final arthritis score was calculated as the sum of scores from all four limbs, which were assessed by three independent individuals blinded to the experimental groups.

### Histological analysis

Histological analysis was conducted to determine the extent of joint damage. Mice joint tissues were fixed in 4% paraformaldehyde, decalcified in EDTA solution, embedded in paraffin, and sectioned. The sections were dewaxed using xylene and dehydrated through an alcohol gradient. Endogenous peroxidase activity was quenched with methanol–3% H_2_O_2_.Sections were stained routinely with hematoxylin–eosin.

### Ethics statement

Ad-hMSCs were obtained by liposuction of abdominal subcutaneous fat from patients who had provided prior informed consent, as approved by the Institutional Review Board of Bucheon St. Mary’s Hospital. The procedures for the use of Ad-hMSCs for experimental studies were approved by the Institutional Review Board of Bucheon St. Mary’s Hospital. All experiments were performed in compliance with the Declaration of Helsinki.

### Culture of human adipose tissue-derived MSCs

Adipose tissue was digested with RTase (4 mL/g fat; K-STEM CELL Co., Ltd., Seoul, Korea) for 60 min at 37 °C. The digested tissues were filtered through a 100-μm nylon sieve to remove cellular debris and then centrifuged at 470 × *g* for 5 min. The pellet obtained was resuspended in RCME cell attachment medium (K-STEM CELL) and cultured overnight at 37 °C in a humidified atmosphere with 5% CO_2_. After 24 h, the cultures were washed with phosphate-buffered saline (PBS) to remove nonadherent cells. The medium was changed to RKCM cell growth medium (K-STEM CELL) containing 5% fetal bovine serum (FBS; Invitrogen, Carlsbad, CA, USA). The cells were cultured for 4 days until 90% confluent (passage zero). The cells were expanded for two or three passages and used for experiments.

### Construction of sRAGE vector and transfection

A fragment of the human sRAGE gene was synthesized by GenScript Corporation, with codon optimization for expression in mammalian cells. The sequence was then subcloned into the *Hin*dIII and *Xho*I sites of pcDNA3.1+ (Invitrogen,Life Technologies, Grand Island, NY, USA). The vector constructs were transfected into MSCs using the X-tremeGENE HP Transfection Reagent (Roche, Mannheim, Germany), according to manufacturer’s recommendations.

### Evaluation of MSC differentiation

Primary ASCs were differentiated using a commercially available differentiation kit (R&D Systems, Lille, France). Briefly, the cells were plated in 24-well plates (adipogenic and osteogenic differentiation) or 15-mL conical tubes (chondrogenic differentiation) and cultured in each differentiation medium. After 1 week (adipogenic differentiation) or 3 weeks (osteoblastogenic and chondrogenic differentiation), cells were fixed and stained with 4′,6-diamidino-2-phenylindole (DAPI), FABP-4, or osteocalcin or aggrecan and visualized by fluorescence microscopy.

### Real-time polymerase chain reaction (PCR)

Messenger RNA (mRNA) was extracted using TRI Reagent (Molecular Research Center, Inc., Cincinnati, OH, USA) according to the manufacturer’s instructions. cDNA was synthesized using a SuperScript Reverse Transcription system (Takara). A Light-Cycler 2.0 instrument (software version 4.0; Roche Diagnostics) was used for PCR amplification. All reactions were performed using the LightCycler FastStart DNA Master SYBR Green I mix (Takara) following the manufacturer’s instructions. The primers used to amplify the mouse genes were as follows: *VEGF*, 5′-CCA-TGA-ACT-TTC-TGC-TGT-CTT-3′ (sense) and 5′-ATCG-CAT-CAG-GGG-CAC-ACA-G-3′ (antisense); *IL-1β*, 5′- TCG-TTA-TCC-CAT-GTG-TCG-AA-3′ (sense) and 5′- GGA-CAA-GCT-GAG-GAA-GAT-GC-3′ (antisense); *IL-6*, 5′- AAT-TCG-GTA-CAT-CCT-CGA-CGG-3′ (sense) and 5′- GGT-TGT-TTT-CTG-CCA-GTG-CC-3′ (antisense); *IL-10*, 5′-TTG-CCT-GGT-CCT-CCT-GAC-TG-3′ (sense) and 5′-GAT-GTC-TGG-GTC-TTG-GTT-CT-3′ (antisense); *HMGB-1*, 5′- GAT-CCC-AAT-GCA-CCC-AAG-AG-3′ (sense) and 5′-TTC-GCA-ACA-TCA-CCA-ATG-A-3′ (antisense); *TGF-β*, 5′-TGC-GGC-AGC-TGT-ACA-TTG-A-3′ and 5′-TGG-TTG-TAC-AGG-GCC-AGG-A-3′ (antisense); *IDO*, 5′-TTT-GGG-TCT-TCC-CAG-AAC-C-3′ (sense) and 5′-GCG-CTG-TTG-GAA-ATA-GCT-TC-3′ (antisense); *HGF*, 5′- CCA-CCA-TAA-TCC-CCC-TCA-CA-3′ (sense) and 5′- GGC-TGG-GGC-TAC-ACT-GGA-TT-3′ (antisense); *CXCR* 1, 5′-GCA-CGA-ACA-GAA-GCT-TTA-T-3′ (sense) and 5′-CTG-AGC-CCC-AAG-TGG-AAC-GA-3′ (antisense); *CXCR4*, 5′-ATC-CCT-GCC-CTC-CTG-CTG-ACT-ATT-C-3′ (sense) and 5′-GAG-GGC-CTT-GCG-CTT-CTG-GTG-3′ (antisense);*CCR1*, 5′-TCC-ATG-CTG-TGC-CAA-GAG-TCA-3′ (sense) and 5′-ACC-ATA-GGA-GGC-CAA-CCC-AAA-ATA-3′ (antisense); *CCR3*, 5′-GGT-TCA-TGC-AGC-AGT-GGG-AGT-AC-3′ (sense) and 5′-TTT-GTC-ATC-ATG-GCG-GTG-TTT-TTC-3′ (antisense); *CCR4*, 5′-GGA-TTA-AGG-CAG-CAG-TGA-ACA-AAA-G-3′ (sense) and 5′-GGA-TTA-AGG-CAG-CAG-TGA-ACA-AAA-G-3′ (antisense); *CCR7*, 5′-GCC-GAG-ACC-ACC-ACC-ACC-TT-3′ (sense) and 5′-AGT-CAT-TGC-ATC-TGC-TCC-CTA-TCC-3′ (antisense). All mRNA levels were normalized to β-actin levels.

### Measurement of cytokine and IgG levels

The concentrations of VEGF, IL-1β, IL-6, RAGE, and HMGB-1 (ABNOVA Corp., Taiwan) in culture supernatants and serum samples were measured using a sandwich enzyme-linked immunosorbent assay (ELISA) (DuoSet; R&D Systems). Serum levels of IgG, IgG1, and IgG3 antibodies were measured using a commercially available ELISA kit (Bethyl Laboratories, Montgomery, TX, USA).

### Western blot analysis

Proteins were separated by sodium dodecyl sulfate polyacrylamide gel electrophoresis (SDS-PAGE) and transferred to nitrocellulose membranes (Amersham Pharmacia Biotech, Buckinghamshire, UK). The membranes were probed with primary antibodies against RAGE and HMGB-1 (Santa Cruz Biotechnology, Santa Cruz, CA, USA) or β-actin (Sigma, St Louis, MO, USA).

### Transwell migration assay

Migration assays were performed in a 24-well Transwell unit with 8-μm pores (Corning Costar, Cambridge, MA, USA). MSCs or sRAGE-MSCs were seeded at a density of 6 × 10^5 ^cells/mL in 100 μL of medium (α-minimal essential medium +1% FBS) in the upper chamber of the Transwell assembly. The lower chamber contained 600 μL of medium with 30 ng/mL SDF-1α (PeproTech, Rocky Hill, NJ, USA). After incubation at 37 °C in 5% CO_2_ for 10 h, the upper surface of the membrane was scraped gently to remove nonmigrating cells and washed with PBS. The membrane was then fixed in 4% paraformaldehyde for 15 min and stained with 0.5% crystal violet for 10 min.

### Confocal microscopy and immunostaining

Tissues were obtained 42 days after CII immunization, snap-frozen in liquid nitrogen, and stored at −80 °C. Tissue cryosections (7 μm thick) were fixed in 4% (v/v) paraformaldehyde and stained using fluorescein isothiocyanate (FITC)-, phycoerythrin-, PerCP–Cy5.5-, or allophycocyanin-conjugated monoclonal antibodies to IL-10, CD4, CD25, IL-17, Foxp3, pSTAT-3 (Y705 and S727), pSTAT-5 (Y694) (all from eBioscience, San Diego, CA, USA), IDO (BD Biosciences, San Jose, CA, USA), and DAPI. After overnight incubation at 4 °C, the stained sections were visualized by confocal microscopy (LSM 510 Meta; Zeiss, Göttingen, Germany).

### Flow cytometry

Totalsplenocyteswere obtained from aseptically collected mousespleen described previously^74^. The cells were harvested in Roswell Park Memorial Institute (RPMI)-1640 medium supplemented with 5% fetal bovine serum. Mononuclear cells (1 × 10^6^) were cultured without treatment as a negative control or with anti-CD3 and anti-CD28 (BD Pharmingen) for 3 days. The cells were stained with various combinations of fluorescence-tagged antibodies against CD4, CD25, IFN-γ (BD Biosciences), Foxp3, and IL-17 (eBioscience). Before intracellular staining, cells were restimulated for 4 h with phorbol myristate acetate (25 ng/mL) and ionomycin (250 ng/mL) in the presence of GolgiSTOP (BD Biosciences). Intracellular staining was performed using a kit (eBioscience) following the manufacturer’s protocol. Stained cells were analyzed on a FACSCalibur apparatus (BD Biosciences).

### Immunohistochemistry

Immunohistochemistry was performed using a VECTASTAIN ABC kit (Vector Laboratories, Burlingame, CA, USA). Tissue sections were incubated overnight at 4 °C with the primary antibodies against RAGE, HMGB-1, and IL-17, probed with a biotinylated secondary antibody, and then stained with a streptavidin–peroxidase complex for 1 h. The final color product was developed using DAB chromogen (Dako, Carpinteria, CA, USA).

### Statistical analysis

The data were analyzed using IBM SPSS Statistics 20 for Windows (IBM Corp., Armonk, NY, USA). One-way analysis of variance followed by Bonferroni’s post hoc test was used to compare differences between three groups. The Mann–Whitney *U* test was used to compare numerical data between two groups. *P* < 0.05 was accepted as significant. Data are presented as mean ± standard deviation (s.d.).

## Additional Information

**How to cite this article**: Park, M.-J. *et al.* Overexpression of soluble RAGE in mesenchymal stem cells enhances their immunoregulatory potential for cellular therapy in autoimmune arthritis. *Sci. Rep.* **6**, 35933; doi: 10.1038/srep35933 (2016).

**Publisher’s note**: Springer Nature remains neutral with regard to jurisdictional claims in published maps and institutional affiliations.
